# Identification of an ancestral haplotype in the mitochondrial phylogeny of the ovine haplogroup B

**DOI:** 10.7717/peerj.7895

**Published:** 2019-10-22

**Authors:** Paolo Mereu, Monica Pirastru, Mario Barbato, Valentina Satta, Eleftherios Hadjisterkotis, Laura Manca, Salvatore Naitana, Giovanni G. Leoni

**Affiliations:** 1 Department of Biomedical Sciences, Sassari University, Sassari, Italy; 2 Department of Animal Sciences, Nutrition and Food, La Cattolica University, Piacenza, Italy; 3 Department of Veterinary Medicine, Sassari University, Sassari, Italy; 4 Ministry of Agriculture, Rural Development and Environment, Nicosia, Cyprus

**Keywords:** Molecular dating, Taxonomy, Mitochondrial haplogroups, Mitogenome, Ovis, Philogeny, Molecular evolution

## Abstract

**Background:**

European mouflon (*Ovis orientalis musimon*) has been reintroduced in mainland Europe since the 18th-century sourcing from the Sardinian and Corsican autochthonous mouflon populations. The European mouflon is currently considered the feral descendent of the Asian mouflon (*O. orientalis*), and the result of first wave of sheep domestication occurred 11,000 years ago in the Fertile Crescent, and brought to Corsica and Sardinia ca. 6,000 years ago, where they still live as autochthonous populations. However, this phylogeny is based on mitogenome sequences of European mouflon individuals exclusively.

**Methods:**

We sequenced the first complete mtDNA of the long-time isolated Sardinian mouflon and compared it with several ovine homologous sequences, including mouflon from mainland Europe and samples representative of the five known mitochondrial domestic sheep haplogroups. We applied Bayesian inference, Maximum Likelihood and Integer Neighbour-Joining network methods and provided a robust, fully-resolved phylogeny with strong statistical support for all nodes.

**Results:**

We identified an early split (110,000 years ago) of the Sardinian mouflon haplotype from both sheep and mainland European mouflon belonging to haplogroup B, the latter two sharing a more recent common ancestor (80,000 years ago). Further, the Sardinian mouflon sequence we generated had the largest genetic distance from domestic sheep haplogroups (0.0136 ± 0.004) among mouflon species. Our results suggest the Sardinian mouflon haplotype as the most ancestral in the HPG-B lineage, hence partially redrawing the known phylogeny of the genus *Ovis*.

## Introduction

Sheep domestication took place in the Near East sometime between the Neolithic and the Upper Palaeolithic (Sanna et al., 2015; Zeder, 2008) and contributed to humans’ new survival strategies based on hunting-gathering and farming (Clutton-Brock, 1999). To date, the Asian mouflon (*Ovis orientalis*) is considered the closest extant species to the ancestor of the domestic sheep (*O. aries*) (Hiendleder et al., 2002; Zohary, Tchernov & Horwitz, 1998), while the European mouflon (*O. o. musimon*) is thought to be a relic of the first domesticated ovine readapted to feral life rather than a wild sheep (Chessa et al., 2009; Poplin, 1979; Vigne, 1988).

Mouflon are free-ranging sheep currently present in the Middle East area (Asian mouflon), in several countries of mainland Europe and in the Mediterranean islands of Sardinia and Corsica (European mouflon). Although geographically included within the European mouflon range, the Cypriot mouflon (*O. o. ophion*) appears to be more genetically related to the Asian mouflon (Sanna et al., 2015). Based on zooarchaeological and molecular data, the first domesticated sheep were introduced ∼12,000 years ago (YA) from the Near East to Europe via the Mediterranean Sea and East European countries following the spread of agriculture, and adapted to the widest range of environmental conditions (Barbato et al., 2017; Chessa et al., 2009; Vigne et al., 2014). Sheep populations from this first wave of migration reached the Mediterranean island of Cyprus (∼10,000 YA), and Corsica and Sardinia (6–7,000 YA) (Agosti et al., 1980; Levine, 1983; Vigne et al., 2014; Zilhao, 2001), where feral populations became established (Guerrini et al., 2015; Poplin, 1979; Zeder, 2008). Around 5,000-6,000 years later, a second wave of domesticated sheep - selected for productive traits such as wool, milk, and meat - replaced the first and less specialized populations (Chessa et al., 2009; Lv et al., 2015; Zeder, 2008). The arrival of this newly domesticated sheep, along with climate change and hunting pressure might have combined to the extent that few or no free-ranging mouflon survived in mainland Europe, whereas they strived in the scarcely populated Mediterranean islands of Sardinia, Corsica and Cyprus (Brooks, Catchpole & Morgan, 2000). Since the 18th century, Corsican and Sardinian mouflon have been used to repopulate several regions of mainland Europe (Damm & Franco, 2014; Turke & Tomiczek, 1982; Uloth, 1972).

The current pattern of differentiation within *Ovis* has been influenced by multiple evolutionary and demographical events such as migration, introgression, mutation, genetic drift, adaptation and isolation, along with natural and/or human-mediated mouflon *x* domestic sheep crossbreeding (Barbato et al., 2017; Lauvergne, Denis & Théret, 1977; Schroder et al., 2016). Records of crossbreeding between feral and domestic sheep are available since Roman times (Pliny the Elder, 1906), and signals of domestic sheep introgression into mouflon have been reported using microsatellite analyses both in mainland and island populations (Guerrini et al., 2015; Lorenzini et al., 2011; Schroder et al., 2016). However, genome-wide scans recorded weak signals of sheep introgression into the majority of European mouflon populations, including the Corsican mouflon. In particular, the sheep introgression in Sardinian mouflon seems to be limited to some reintroduced/managed populations, whereas those populations living in the historical mouflon range appear overall pure (Barbato et al., 2017).

Mitochondrial DNA (mtDNA) sequence analysis is the most convenient and cheapest molecular tool to infer the origin of the species and to estimate levels of genetic diversity into the process of domestication, and is considered the backbone of molecular genetic investigations in livestock (Alvarez et al., 2004; Demirci et al., 2013; Di Lorenzo et al., 2016; Guerrini et al., 2015; Meadows et al., 2007; Meadows, Hiendleder & Kijas, 2011; Pedrosa et al., 2005; Sanna et al., 2015; Tapio et al., 2005; Tapio et al., 2006). To date, five different domestic sheep (*Ovis aries*) mitochondrial haplogroups (HPGs) have been identified (Meadows, Hiendleder & Kijas, 2011). Among them two main clusters can be identified: cluster I which is mostly represented in Asia (HPG-C) and SW-Asia (HPG-E), and cluster II mostly represented in Europe (HPG-B) and Middle East (HPGs A and D) (Sanna et al., 2015). The mainland Europe and Asian mouflon mitogenome sequences show a close relationship with HPG-B sheep (Meadows, Hiendleder & Kijas, 2011), while the Cypriot mouflon is the closest extant species to the ancestor of cluster I (Sanna et al., 2015).

With this work we aimed to investigate the phylogenetic relationship between Sardinian mouflon and its wild, feral, and domestic relatives. We carried out the molecular comparison between Sardinian mouflon and other *Ovis* mitogenome sequences—including mouflon from mainland Europe—and the five mitochondrial domestic sheep haplogroups currently identified.

## Materials & Methods

### Sample collection

All the animal procedures were performed in strict accordance with the guidelines of the Ethics Committee of Sassari University, Italy, which also approved this study. The biological samples were obtained from a female mouflon which died for natural causes, belonging to a feral population living in the Montes forest (40°12′19.08″–N 9°21′16.02″E), located within the Gennargentu national park in the mountainous Central-Eastern part of Sardinia. This population remained isolated from contacts with domestic species up to the present day and is considered, from a historical point of view, one of the oldest Sardinian mouflon colonies.

### DNA extraction, amplification and sequencing

Genomic DNA was extracted from muscle using the GenElute Mammalian genomic DNA miniprep kit (Sigma-Aldrich) according to the manufacturer’s protocol. Sample quality and DNA concentration were determined via spectrophotometry using a ND-8000 (NanoDrop Technologies, Thermo Fisher Scientific Inc., Wilmington, DE, USA). Total yield of DNA extracted was ∼21 µg (DNA conc. 125–150 ng/µl with an elution volume of 150 µl).

The 21 pairs of primers reported in Sanna et al. (Sanna et al., 2015) were used to amplify overlapping fragments spanning the whole mitogenome of the Sardinian mouflon.

PCR products were purified by electrophoresis on a 1.2% agarose gel using the Montage™ DNA Gel extraction kit (Millipore) and then sequenced with each of the PCR primers on an ABI 3130 sequencer. Sequences obtained were aligned, edited and assembled using Clustal X2 (Larkin et al., 2007) and the software CAP3 Assembly Program (Huang & Madan, 1999) to generate the complete mitogenome sequence. The protein-coding genes were determined by open reading frame finder implemented at the NCBI website with the vertebrate mitochondrial genetic code, and then finally confirmed by sequence comparisons with the reported *O. aries* mitogenome (GB# NC_001941).

The physical map of the Sardinian mouflon mitogenome was generated by means of OGDraw 1.2 (Lohse et al., 2013).

### Homologous sequences assembly, dataset composition and phylogenetic analyses

Three data set including 69 D-loop (Table S1), 90 CytB (Table S2) and 29 complete mitogenome (Table S3) sequences, respectively, were assembled for phylogenetic analyses.

For the complete mitogenome, the comparisons were performed on both the entire mtDNA sequences and 28H marker encompassing the concatenated sequences of the 12 protein-coding genes, 14 transfer RNA genes and two ribosomal RNA genes, all located on the H-strand, as previously described (Sanna et al., 2015). We produced a joined dataset using the complete mitogenome sequence of a Sardinian mouflon from the Montes area obtained in this study, plus 27 sequences collected from GenBank, and the mitogenome sequence of an Asian mouflon (KF938360), recently characterized (Lv et al., 2015) (Table S3). Ingroup taxa included 17 *Ovis* species, the *Moschus moschiferus* was selected to root the phylogenetic trees while the remaining 11 outgroup sequences were used as calibration points for the divergence time estimations.

Sequences were aligned using the Clustal X2 software (Larkin et al., 2007). DNA sequence variation parameters were estimated using the software package DnaSP 5.10 (Librado & Rozas, 2009). The best-fit model for each dataset was selected using MEGA 7.0.14 (Kumar, Stecher & Tamura, 2016). The general time-reversible model with discrete gamma distribution and invariant sites (GTR+G+I) had the highest score for both the D-loop and the complete mitogenome sequences, while GTR+G was the best for the CytB data set.

Phylogenetic relationships among individuals were investigated by means of Bayesian Inference (BI) and Maximum Likelihood (ML) analyses. MrBayes 3.2.4 (Ronquist et al., 2012) was used for BI and MEGA 7.0.14 (Kumar, Stecher & Tamura, 2016) for ML tree reconstruction. Analyses for BI tree reconstruction were carried out for five million generations and the trees were sampled every 10 generations. The reliability of each branch in ML tree was estimated by bootstrapping (10,000 replications)

The TCS network analysis was performed using the software Pop ART 1.7 (Leigh & Bryant, 2015), to identify possible disconnections between groups of individuals, and infer the genetic relationships among the haplotypes.

Divergence time estimation was inferred by the complete mitogenome sequences analysis by assuming seven different calibration points (CPs) based on fossil records providing ages for nodes inside Bovidae (Bibi, 2013) as previously described (Sanna et al., 2015).

## Results

### Mitogenome characterization

We sequenced the complete mtDNA sequence of a Sardinian mouflon individual and deposited it in GenBank (MG489885). The obtained 16,618 bp long molecule showed a percentage composition in bases of the L-strand of 33.7% A, 25.8% C, 13.1% G, and 27.4% T. Overall, we detected 13 protein coding genes, 12S and 16S ribosomal RNA, 22 transfer RNA genes and a D-loop region, according to the molecule gene content of the other *Ovis* species previously reported (Hiendleder et al., 1998; Lv et al., 2015; Meadows, Hiendleder & Kijas, 2011; Sanna et al., 2015). The structural organization of Sardinian mouflon mitogenome and the location of the various features are displayed in Fig. S1 and summarized in Table 1.

**Table 1:** Organization of the Sardinian mouflon mitogenome.

Gene	Location	Size	Start codon	Stop codon	3′ spacer/overlap
tRNA-Phe	1-68	68			
12S rRNA	69-1,027	959			
tRNA-Val	1,028-1,094	67			
16S rRNA	1,095-2,669	1,575			
tRNA-Leu	2,670-2,744	75			AA-base spacer
NADH 1	2,747-3,701	955	ATG	Taa^†^	A-base spacer
tRNA-Ile	3,703-3,771	69			3-base overlap
tRNA-Gln (L)	3,769-3,840	72			AT-base spacer
tRNA-Met	3,843-3,911	69			
NADH 2	3,912-4,953	1,042	ATA	Taa^†^	
tRNA-Trp	4,954-5,020	67			A-base spacer
tRNA-Ala (L)	5,022-5,090	69			A-base spacer
tRNA-Asn (L)	5,092-5,164	73			
O _L_	5,165-5,196	32			
tRNA-Cys (L)	5,197-5,264	68			
tRNA-Tyr (L)	5,265-5,332	68			C-base spacer
COX I	5,334-6,878	1,545	ATG	TAA	2-base overlap
tRNA-Ser (L)	6,877-6,946	70			TAAAC-base spacer
tRNA-Asp	6,952-7,019	68			T-base spacer
COX II	7,021-7,704	684	ATG	TAA	AAT-base spacer
tRNA-Lys	7,708-7,775	68			T-base spacer
ATP 8	7,777-7,977	201	ATG	TAA	40-base overlap
ATP 6	7,938-8,618	681	ATG	TAA	1-base overlap
COX III	8,618-9,401	784	ATG	Taa^†^	
tRNA-Gly	9,402-9,470	69			
NADH 3	9,471-9,816	346	ATA	Taa^†^	A-base spacer
tRNA-Arg	9,818-9,886	69			
NADH 4L	9,887-10,183	297	ATG	TAA	7-base overlap
NADH 4	10,177-11,554	1,378	ATG	Taa^†^	
tRNA-His	11,555-11,623	69			
tRNA-Ser	11,624-11,683	60			A-base spacer
tRNA-Leu	11,685-11,754	70			
NADH 5	11,755-13,575	1,821	ATA	TAA	17-base overlap
NADH 6 (L)	13,559-14,086	528	ATG	TAA	
tRNA-Glu (L)	14,087-14,155	69			ACTA-base spacer
Cyt B	14,160-15,299	1,140	ATG	AGA	CAA-base spacer
tRNA-Thr	15,303-15,372	70			1-base overlap
tRNA-Pro (L)	15,372-15,437	66			
D-loop	15,438-16,618	1,181			

**Notes.**

(L) indicates a gene encoded on the L-strand.

† Incomplete stop signals.

### D-loop and CytB sequences analysis

**Figure 1:**
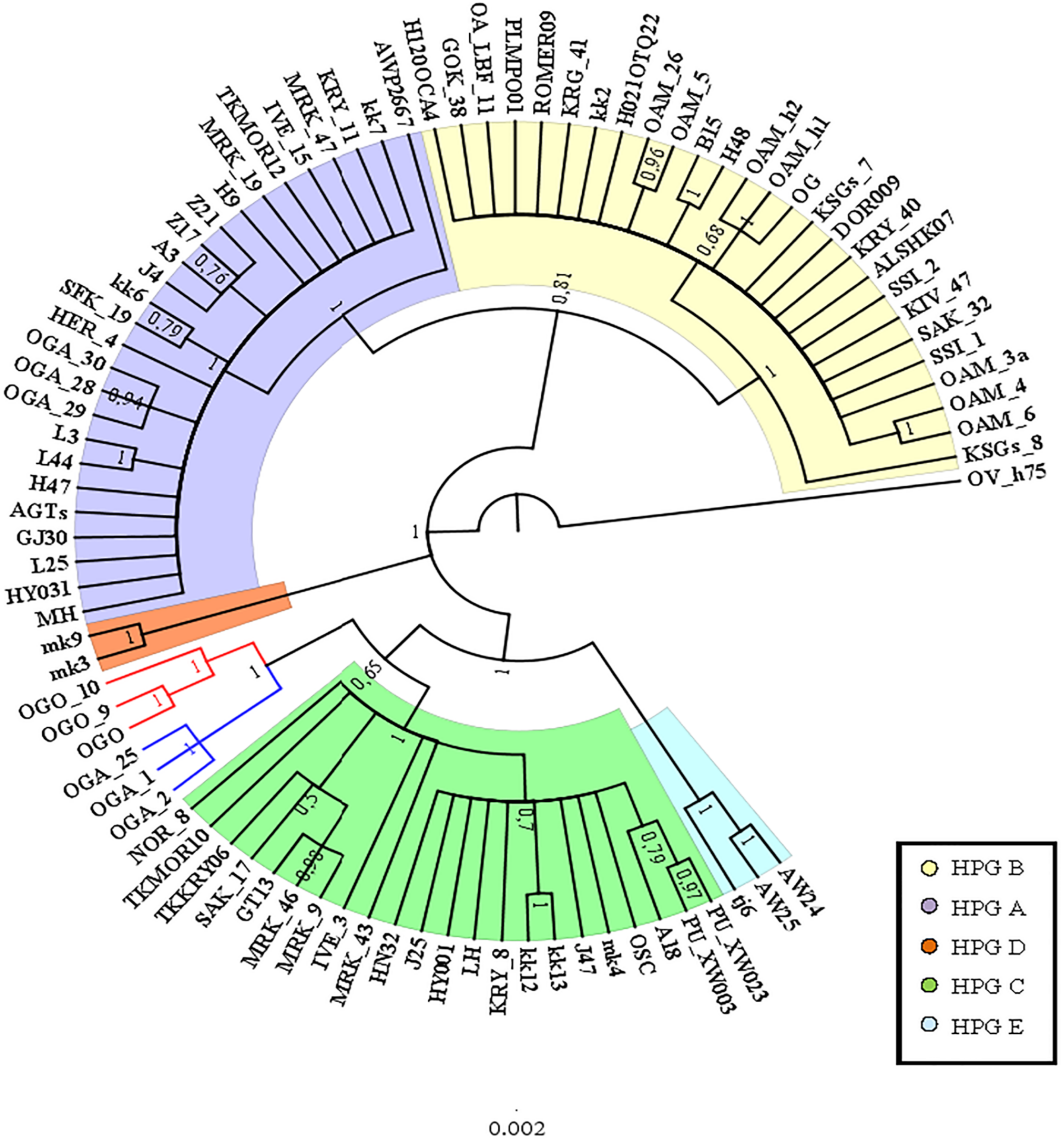
Bayesian rooted tree of the D-loop sequences. Phylogenetic relationships among wild mouflon and domestic sheep based on the D-loop sequences analysis.

A preliminary phylogenetic analysis was performed using the mtDNA D-loop region and the CytB gene as molecular markers. The data set of D-loop region consisted of 69 sequences from domestic sheep and 17 from wild mouflons (Table S1). The urial sequence was selected as outgroup to root the BI tree (Fig. 1).

Five domestic sheep HPGs were detected, accordingly with those described by Meadows, Hiendleder & Kijas (2011) on the entire mitogenome sequence analysis. Three main evolutionary lineages within the sheep/mouflon early radiation gave birth to HPG D, the clade including HPGs A and B, and another consisted of HPGs C and E grouped together with haplotype (Hpt X), respectively. The sequences from wild species were *not uniformly distributed* throughout the HPGs. The European mouflon sequences (OAMs in Table S1) all belong to HPG B where even a sequence from an Asian mouflon (OG) falls.

The OAM sequences were further grouped in three distinct clades. The OAM_3 sequence was not included within any group. The ML analysis (Fig. S2) provided an identical but slightly lower supported tree topology.

**Figure 2:**
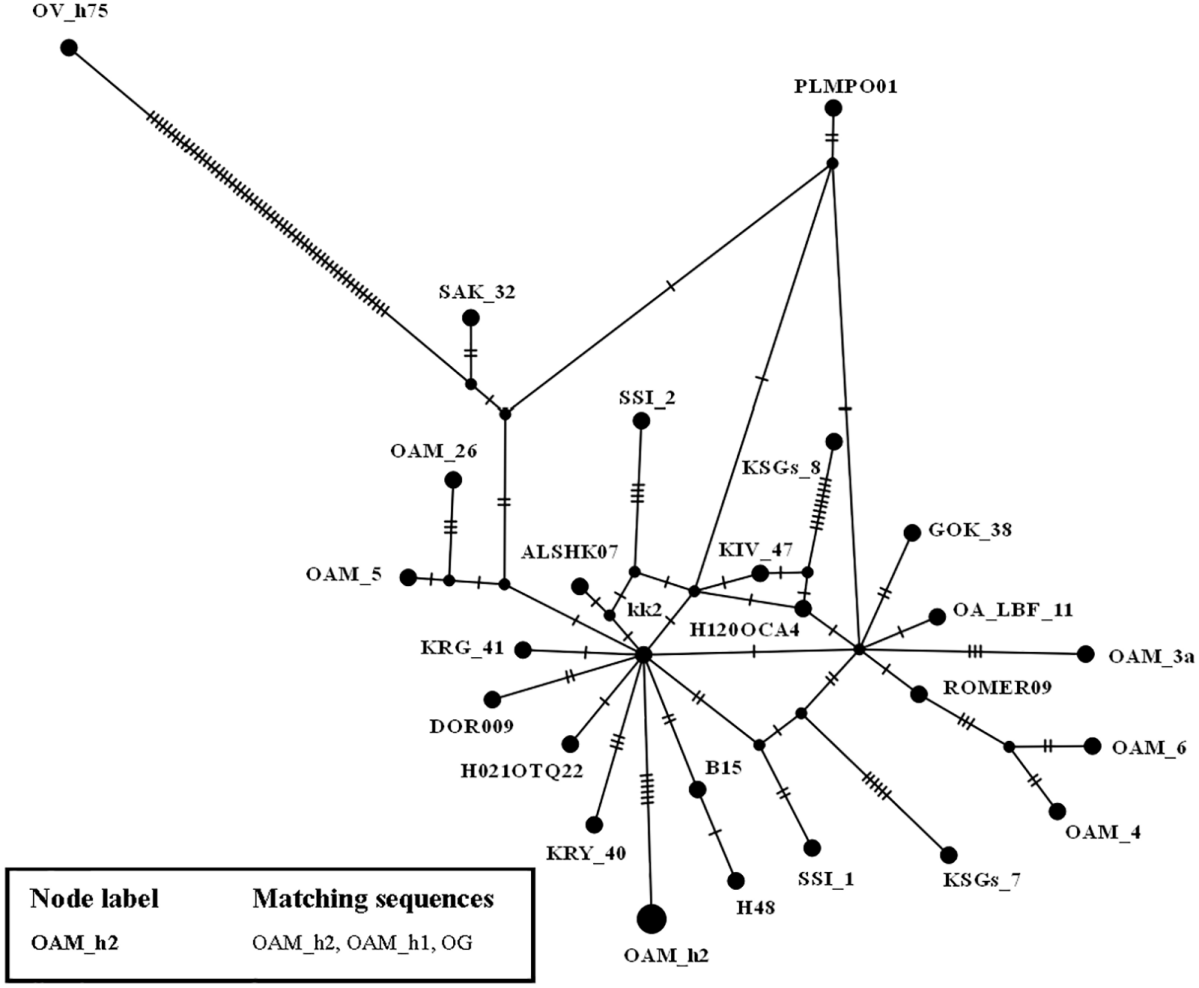
TCS network of the D-loop sequences. TCS network showing the different relationship between the central common ancestor (CCA) kk2 and the Sardinian mouflon sequence, with respect to the German and Asian mouflon sequences.

To further investigate the relationships among wild and domestic species within the HPG B, a TCS network analysis was carried out (Fig. 2).

By contrast to the other European and Asian mouflon sequences the position of the OAM_26 and OAM_5 could support an early split from the urial lineages before the advent of the first domestic sheep species originating from a central common ancestor (kk2 nucleus in Fig. 2).

**Figure 3:**
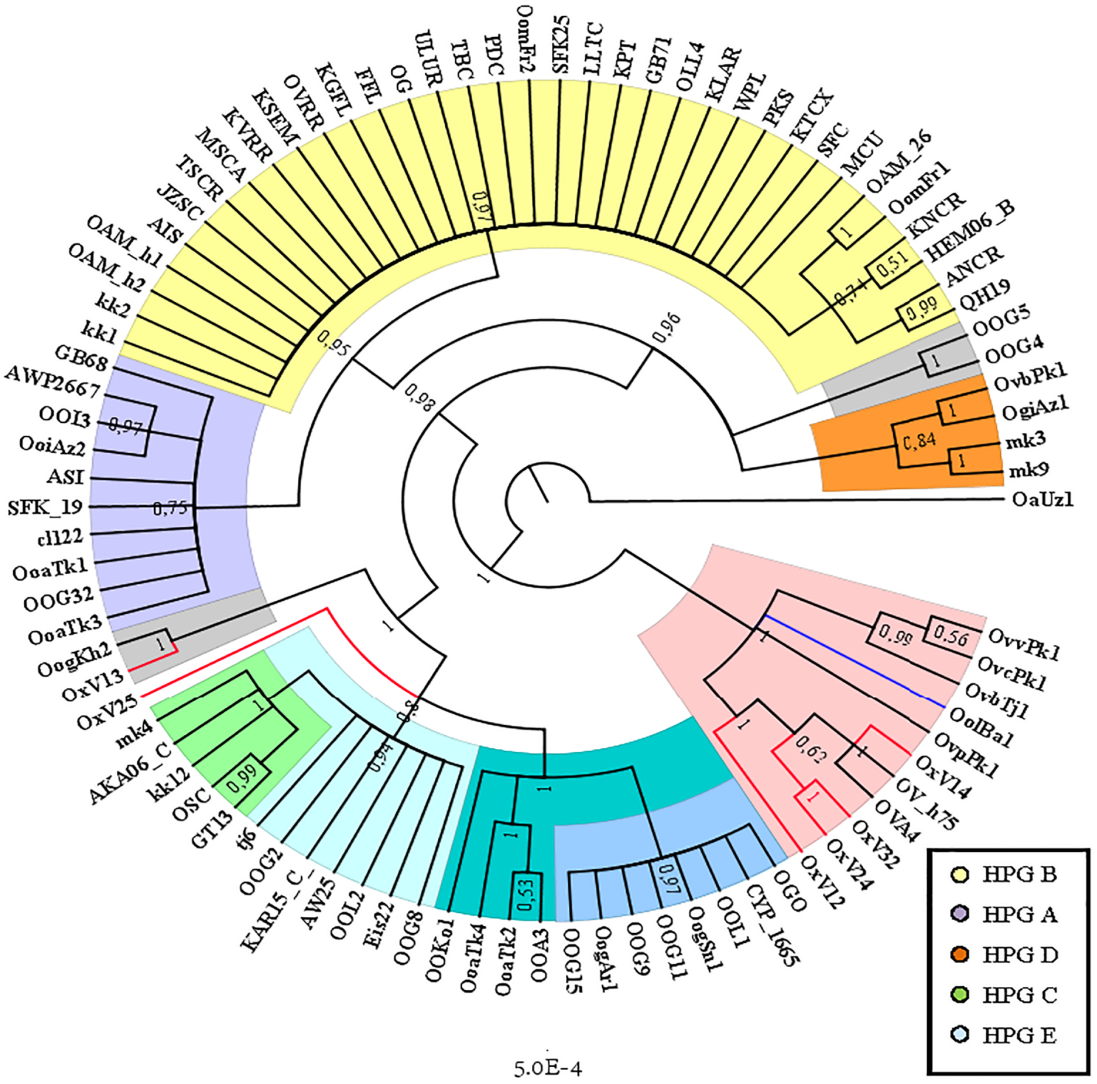
Bayesian rooted tree of the Cyt B sequences. Phylogenetic tree showing the different distribution of Asian and European mouflon sequences among the five know ovine haplogroups.

The Cyt B sequences analysis allowed to significantly increase the number of wild species included in the investigation (Table S2). Indeed, the *O. orientalis* sequences filled the 34.4% of the entire dataset, 6.7% were from hybrid *O. orientalis*/*O. vignei* individuals, 7.8% from *O. vignei* and the remaining 50% belonged to *O. aries*. The OAM_26 sequence was the only one to be selected from the Sardinian group because of its crucial position, together with the OAM_5, in the D-loop analysis (Fig. 2). The BI tree analysis (Fig. 3) evidenced the raise of four mtDNA lineages within the *O. aries*/*O. orientalis* clade: three already detected in the D-loop sequences analysis and a further one originating the OOG4-OOG5 sequences group.

**Figure 4:**
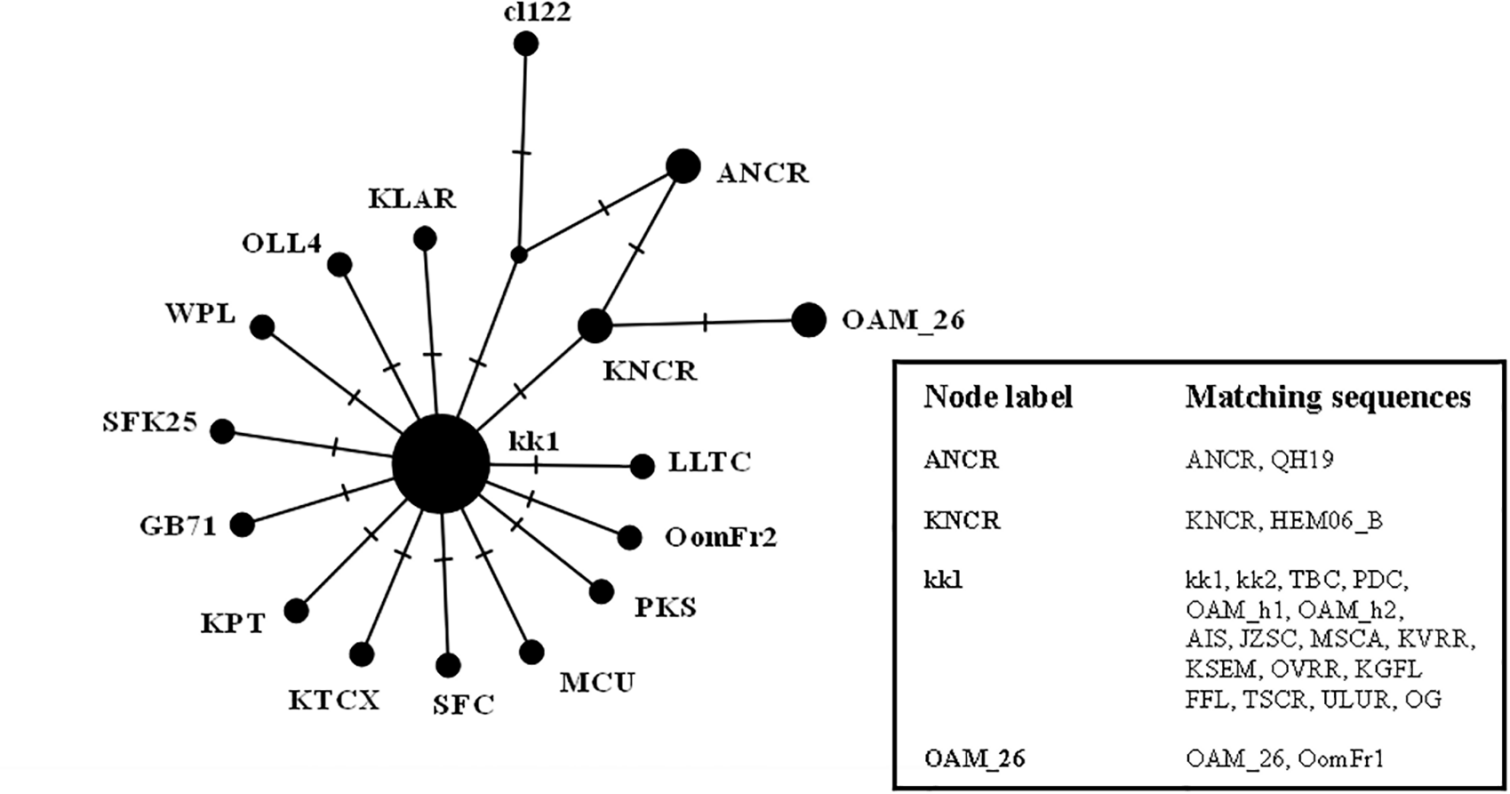
TCS network of the CytB gene sequences. Phylogenetic network highlighting the external position of the Sardinian mouflon sequence OAM_26 with respect to the domestic sheep kk1 nucleus which includes the German (OAM_h1, OAM_h2) and Asian (OG) mouflon sequences.

The results were similar to those obtained in D-loop analysis also including an increased number of wild species: all the European mouflon sequences are grouped within the HPG B, where no Asian mouflon sequence falls, except for the OG one, which given the matrilineal transmission of the mtDNA, could be sampled from a hybrid individual. Moreover, the OAM_26 sequence was found to be not related to sequences from German mouflons, as indicated in the TCS network (Fig. 4).

The ML analysis (Fig. S3) provided an identical but slightly lower supported tree topology.

### Complete mitogenome phylogeny and molecular dating

**Figure 5:**
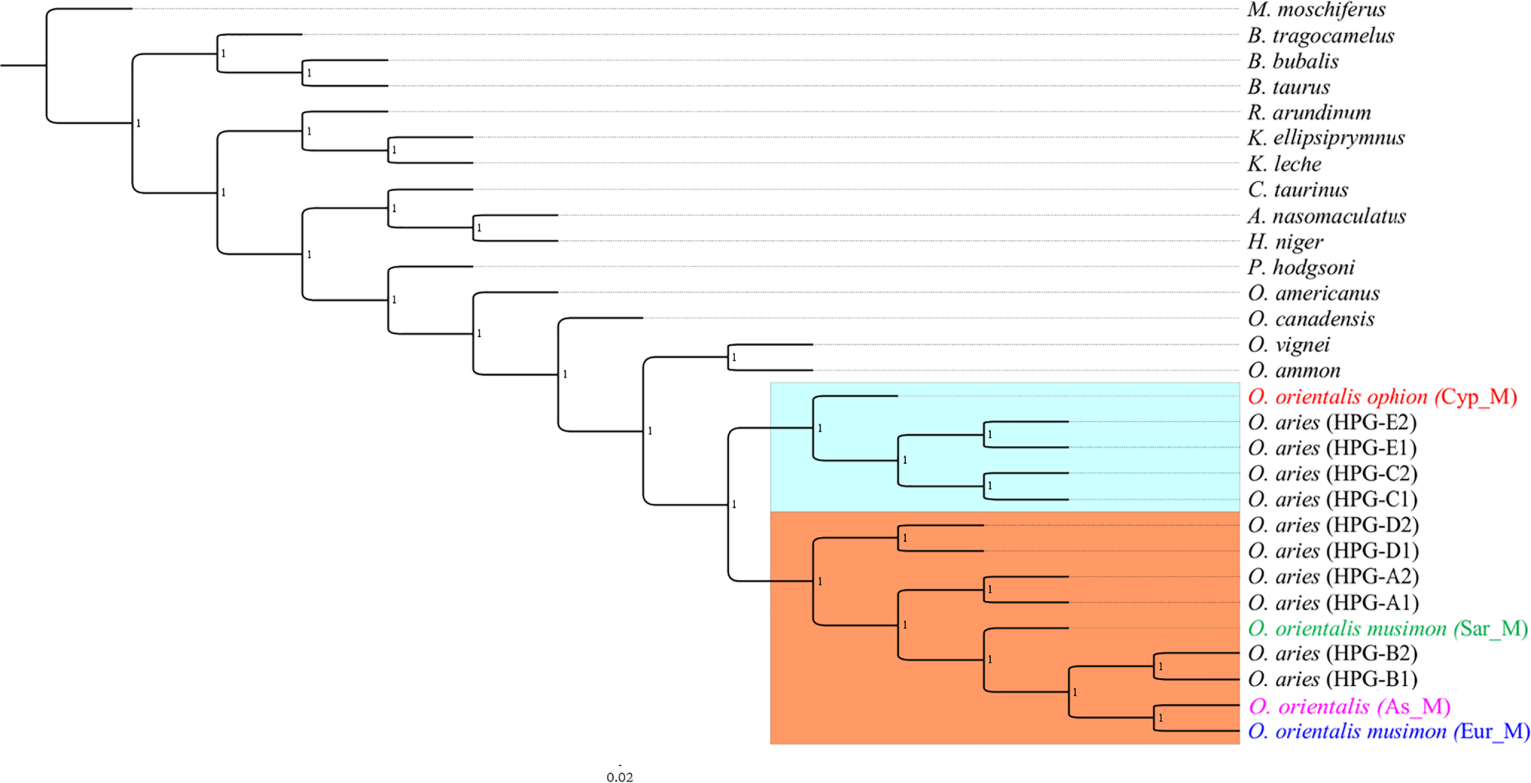
Bayesian rooted tree of complete mitogenome sequences. Phylogenetic tree inferred by the analysis (five million generations) of the entire mtDNA sequences showing the early split of the Sardinian mouflon from the HPG-B clade. All nodes are supported with maximum statistical values (PP = 1). The tree was rooted by using *Moschus moschiferus* mtDNA genome sequence. The scale bar refers to the number of substitutions per site.

Phylogeny on whole mitogenome was inferred using a Sardinian mouflon sequence from this study (MG489885), the dataset from Sanna et al. (2015), and an Asian mouflon sequence collected from GenBank (KF938360) (Table S3). The BI tree analysis performed on both the whole mitogenome and the 28H dataset supported the same relationships among species as confirmed by the identical tree topologies obtained (Fig. 5).

The ML trees (Fig. S4) obtained from the two datasets were comparable with BI results, albeit showing slightly lower supported tree topology. We identified two main clusters in the sheep/mouflon radiation (Prob ≥ 0.99) (Fig. 5).

Cluster II was characterized by two main groups: a first one including HPG-D, and a second one grouping HPGs A and B, and the Sardinian, mainland Europe and Asian mouflon. The Sardinian mouflon haplotype split before the rise of HPG-B sheep, Asian and mainland Europe mouflon, the latter two sharing a most recent common ancestor (MRCA).

**Figure 6:**
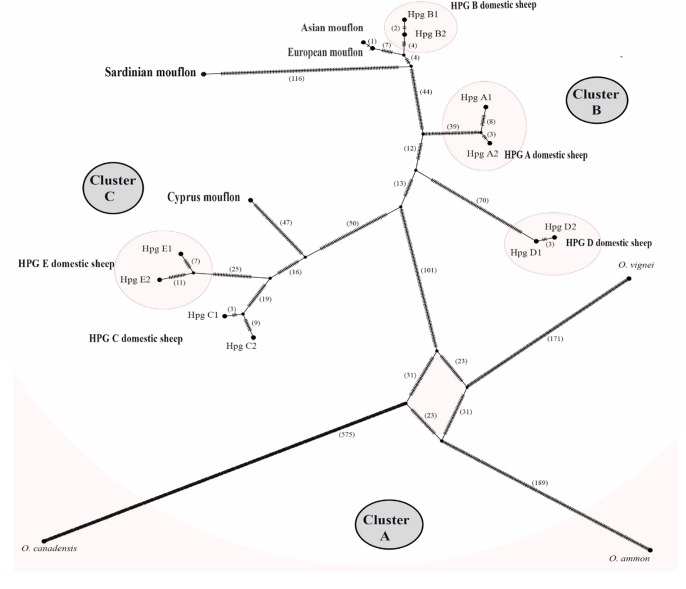
TCS network of the whole mitogenome sequences. Haplotype network showing three main cluster within the *Ovis* species radiation. Dashes represent mutational steps between nodes. The number of mutations between two nodes is indicated in parenthesis near the line linking them. Cluster names are in capital letters within circles.

The TCS network analysis (Fig. 6) of the whole *Ovis* mitogenome sequences retrieved three main disconnected clusters within the *Ovis* genus (Figs. 6A–6C).

Cluster A was exclusive of the wild sheep species included in the network analyses (argali, bighorn and urial). Cluster B encompassed HPGs D, A and B domestic sheep, along with Sardinian, mainland Europe and Asian mouflon sequences. Cluster C grouped the Cypriot mouflon and HPGs C and E domestic sheep. Within cluster B, the mainland Europe and Asian mouflon sequences constituted a sub-clade with HPG-B domestic sheep, while the Sardinian mouflon held an external position, showing a longer evolutionary process as confirmed by the high number of nucleotide substitution in its own lineage. A similar result was observed in the relationship among species within Cluster C, where a sub-clade encompassing HPGs C and E was detected, and the Cypriot mouflon held an external position.

The divergence time estimations dated back the early domestic sheep radiation to ∼410,000 YA, while the speciation events which gave rise to Cluster I and II dated 190,000 and 330,000 YA, respectively. Within Cluster II, the Sardinian mouflon haplotype split about 110,000 YA from the sub-clade including HPG-B sheep and mainland Europe mouflon sequence, which shared a common ancestor 8,000 YA. According to the results provided from the previous analyses carried out on the D-loop e CytB sequences, the KF938360 sequence was grouped within this same sub-clade, something that confirms the hypothesis that it may have been collected from a hybrid individual.

### Genetic variability estimation based on mitogenome sequence analysis

Among the 17 complete mitogenomes belonging to five species within the *Ovis* genus, 17 haplotypes defined by 1,387 polymorphic sites (S) were found. Overall, high values of total mean haplotype and nucleotide diversity were obtained (*h* = 1 and *π* = 0.016, respectively). A lower value of nucleotide diversity (*π* = 0.008) was found among the *O. aries* individuals.

To quantify the divergence among HPGs, pairwise genetic distances between groups were calculated under the K2P model. The variation rate among sites was modelled with a gamma distribution (shape parameter = 0.64). Among domestic sheep HPGs the average value of genetic differentiation was high at 0.0086 (± 0.0006); the lowest level was observed between HPGs C and E (0.0036 ± 0.0004), closely followed by HPGs A and B (0.006 ± 0.0055). The distance between wild species and domestic sheep HPGs ranged from 0.055 ± 0.0009 (Bighorn *vs* domestic HPGs) to 0.0072 ± 0.004 (European mouflon *vs.* domestic HPGs). Among mouflon species, the Sardinian mouflon showed the largest genetic distance from domestic sheep HPGs (0.0136 ± 0.004), followed by Cypriot (0.0087 ± 0.0028), Asian (0.00723  ± 0.004) and mainland Europe mouflon sequences (0.00717 ± 0.004).

## Discussion

First we investigated the phylogenetic relationship in the dataset using the D-loop region and CytB gene as markers, then the mitogenome of the OAM_26 Sardinian mouflon was fully sequenced, due to its critical position in the previous analyses. Indeed, unlike those from German individuals, the OAM_26 was not grouped within the kk1 nucleus representing the central common ancestor which gave rise to the domestic species (Fig. 4). Furthermore, the OAM_26 sequence, along with the OAM_5 one, was the closest to the outgroup among those from mouflon individuals (Fig. 2), suggesting an early split which predates the origin of the first domestic sheep species. These two sequences were obtained from individuals sampled in one of the mouflon ancestral ranges in Sardinia, where they survived virtually undisturbed thanks to the combination of harsh terrain and ad-hoc conservation policies enforced by the regional government.

The inclusion of a Sardinian mouflon mitogenome sequence in our dataset allowed to shed new light on the relationship between mouflon and domestic sheep belonging to HPG-B.

### The origin of the ovine mitochondrial HPG-B

HPG-B is the most common mitochondrial haplogroup among extant domestic sheep, including the vast majority of cosmopolitan sheep breeds (Hiendleder et al., 1998; Meadows, Hiendleder & Kijas, 2011). The molecular dating we performed shows the Sardinian mouflon haplotype (HPT-B1) stemming about 30,000 years earlier than the HPG-B clade (∼80,000 YA), which includes domestic sheep and mainland Europe mouflons (Fig. 2). We suggest the presence of multiple mitochondrial ovine HPTs populating the Fertile Crescent around the end of the last glacial period, including those identified in the Cypriot, Sardinian (HPT-B1), mainland Europe (HPT-B2), and Asian mouflon. Our results suggest HPT-B1 to be a putative ancestral haplotype of the HPG-B lineage. Among the extant wild species we analysed, HPT-B1was found exclusively in the Sardinian mouflon from the Montes area. However, analyses on an extended dataset comprising more mouflon individuals and populations (e.g., Corsican mouflon) are necessary to confirm this result.

### Mouflon and HPG-B sheep colonization of mainland Europe

Two hypotheses for the predominance of HPG-B in European sheep have been proposed. The most recent and accredited one, which is strongly supported by zooarchaeological and molecular evidences, suggests the predominance of HPG-B in European sheep as the result of the founder effect driven by a subset of founder animals belonging to the HPG-B (Tapio et al., 2006). The second one invokes an independent European sheep domestication event (Ryder, 1981) but there is no zooarchaeological findings to supports this hypothesis.

Our results show that the Sardinian mouflon haplotype (HPT-B1) split 110,000 YA from the mainland Europe mouflon (HPT-B2) and HPG-B sheep (HTP-B3) lineages, the latter sharing a MRCA 80,000 YA (Figs. 2 and 3), confirming the first hypothesis. Likely, the first settlers happened to choose HPT-B1, HPT-B2, and HPT-B3 from a wider pool of haplotypes, and brought them along when they moved from the Near East to Western Europe after the last glaciations (14 to 12,000 YA) (Fu et al., 2016), eventually colonizing Europe, including Corsica and Sardinia (Chessa et al., 2009; Clutton-Brock, 1999). A fascinating hypothesis is that the mutation that modified the fleece from hirsute to woolly—which putatively occurred on HPT-B3 individuals—was key in determining the value of mouflon to their early farmers (Demars et al., 2017). Since the phenotypic traits related to the hair type are not related to mtDNA sequence modifications, the exclusivity of the HPT-B3 in the HPG-B domestic sheep would be the result of co-selection for non-related traits. Since then this trait underwent artificial selection and the economic interest for the mouflon-like ovines quickly decreased, leading the mouflon to survive as feral populations. Alternatively, only the Sardinian and possibly the mainland Europe mouflon haplotypes (HPT-B1 and B2) were included by chance within the first group of individuals moved to Europe from the Neolithic people, as part of what is commonly described as the “first wave of sheep domestication” (Chessa et al., 2009). Successively, individuals harbouring the HPT-B3—and selected for new and favourable secondary traits—arrived to Europe (second wave of domestication) and replaced the resident ovine populations (Chessa et al., 2009; Hiendleder et al., 1998; Poplin, 1979).

## Conclusion

Our data suggest that over the millennia following the early introduction of mouflon in Europe HPT-B1 survived in Sardinia exclusively, probably due to a combination of genetic drift, founder effect, human hunting (Bon et al., 1993), and/or wolf predation (Musiani et al., 2003), whereas HPT-B2 and HPT-B3 persisted in mainland Europe. After its extinction from mainland Europe, mouflon was reintroduced in several European regions since the 18th century, sourcing from both the Sardinian and the Corsican stocks (Guerrini et al., 2015; Turke & Tomiczek, 1982). Given the great genetic distance we detected between the extant Sardinian and mainland Europe mouflon, we speculate that the Corsican mouflon could have strongly impacted the re-colonization of continental Europe areas and therefore the mainland Europe mouflon mtDNA haplotype could be of Corsican ancestry. However, further analyses including Corsican mouflon mitogenome sequences are necessary to confirm this hypothesis. Overall, our results suggest that HPT-B1 occupies a crucial phylogenetic position in the raise of the current HPG-B and that it has been maintained only in the Sardinian mtDNA pool. This molecular evidence provides implications for more accurate management and conservation programs and confirms the role of the Mediterranean islands as biodiversity reservoir, as in the case of the Cypriot mouflon (Sanna et al., 2015), the European griffon vulture (Mereu et al., 2017), Sardinian miniature horses (Morelli et al., 2014), and the Sardinian red deer (Doan et al., 2017).

##  Supplemental Information

10.7717/peerj.7895/supp-1 Table S1 D-loop sequences included in the phylogenetic analysis

10.7717/peerj.7895/supp-2 Table S2 CytB sequences included in the phylogenetic analysis

10.7717/peerj.7895/supp-3 Table S3 Complete mtDNA sequences included in the phylogenetic analysis

10.7717/peerj.7895/supp-4 Figure S1 Structural organization of the Sardinian mouflon mitogenome Arrows indicate the reading frame orientation of each strand. Different colours indicate functional gene categories as indicated in the legend. The inner circle is a graph depicting GC content across the genome (dark gray bars = percentage GC).

10.7717/peerj.7895/supp-5 Figure S2 Maximum likelihood analysis tree of the wild mouflon and domestic sheep D-loop sequences

10.7717/peerj.7895/supp-6 Figure S3 Maximum likelihood analysis tree of the Asian and European mouflon Cyt B sequences among the five know ovine haplogroups

10.7717/peerj.7895/supp-7 Figure S4 Rooted tree obtained by Maximum likelihood for whole mitogenome sequences All nodes representing the domestic sheep and mouflon species radiation are supported with bootstrap values ≥ 0.96. The tree was rooted using *Moschus moschiferus* mtDNA genome

## Additional Information and Declarations

### Field Study Permissions

The following information was supplied relating to field study approvals (i.e., approving body and any reference numbers): A written permit has been required for samples collection. There is a verbal agreement between the regional agency FORESTAS (represented by Dr. Giovanni Bassu, the head of the Montes FORESTAS reserve) and our research group. All samples obtained from animals found dead from natural causes within the park boundaries are sent to us for research purposes.

### Data Availability

The following information was supplied regarding data availability: Data is available at GenBank: MG489885.
