# Supplementary material for: Identification of an ancestral haplotype in the mitochondrial phylogeny of the ovine haplogroup B
Source: PeerJ. 2019 Oct 22;7:e7895. doi: 10.7717/peerj.7895 (PMC6814065; doi:10.7717/peerj.7895)
Supplement: Table S1 [file peerj-07-7895-s001.docx]

| ACCESSION NUMBER | CODE | SPECIES | BREED | ORIGIN |
| --- | --- | --- | --- | --- |
| KX344135 | A3 | *O. aries* |  | China |
| AY091498 | AGTs | *O. aries* | Gizarr | Tadjikistan |
| JX235859 | AWP2667 | *O. aries* | Awassi | Pakistan |
| DQ903235 | GJ30 | *O. aries* |  | China |
| KX344226 | H47 | *O. aries* |  | China |
| KX344188 | H9 | *O. aries* |  | China |
| KF677119 | HER_4 | *O. aries* | Herik | Turkey |
| DQ460459 | HY031 | *O. aries* |  | China? |
| KF677100 | IVE_15 | *O. aries* | Ivesi | Turkey |
| KX344328 | J4 | *O. aries* |  | China |
| DQ852286 | kk6 | *O. aries* | Karakas | Turkey |
| DQ852287 | kk7 | *O. aries* | Karakas | Turkey |
| KF677029 | KRY_11 | *O. aries* | Karayaka | Turkey |
| KX344254 | L25 | *O. aries* |  | China |
| KX344232 | L3 | *O. aries* |  | China |
| KX344273 | L44 | *O. aries* |  | China |
| JQ804992 | MH | *O. aries* |  | China? |
| KF677246 | MRK_19 | *O. aries* | Morkaraman | Turkey |
| KF677262 | MRK_47 | *O. aries* | Morkaraman | Turkey |
| KU681221 | SFK_19 | *O. aries* | Suffolk sheep | China |
| JN574164 | TKMOR12 | *O. aries* | Morkaraman | Turkey |
| KX344386 | Z17 | *O. aries* |  | China |
| KX344390 | Z21 | *O. aries* |  | China |
| JN573907 | ALSHK07 | *O. aries* | Shkodrane | Albania |
| KX344294 | B15 | *O. aries* |  | China |
| AY829381 | DOR009 | *O. aries* | Polled Dorset | China |
| KF677067 | GOK_38 | *O. aries* | Gokceada | Turkey |
| DQ491596 | H021OTQ22 | *O. aries* | Churra da Terra Quente | Portugal |
| DQ491695 | H120OCA4 | *O. aries* | Campanica | Portugal |
| KX344227 | H48 | *O. aries* |  | China |
| HM236177 | kk2 | *O. aries* | Karakas | Turkey |
| KF677149 | KRG_41 | *O. aries* | Karagul | Turkey |
| KF677036 | KRY_40 | *O. aries* | Karayaka | Turkey |
| KR011777 | KSG_7 | *O. aries* | Chios sheep | Greece |
| KR011778 | KSG_8 | *O. aries* | Chios sheep | Greece |
| KT879011 | OA_LBF_11 | *O. aries* | Latxa Black Face | Spain |
| JN574048 | PLMPO01 | *O. aries* | Polish Merino | Poland |
| JN574076 | ROMER09 | *O. aries* | Transylvanian Merino | Romania |
| KF677179 | SAK_32 | *O. aries* | Sakiz | Turkey |
| KR011770 | SSI_1 | *O. aries* | Sardinian sheep | Sardinia (Italy) |
| KR011771 | SSI_2 | *O. aries* | Sardinian sheep | Sardinia (Italy) |
| KF677236 | KIV_47 | *O. aries* | Kiviricik | Turkey |
| KX344150 | A18 | *O. aries* |  | China |
| KU681187 | GT13 | *O. aries* | Tibetan sheep | China |
| DQ903251 | HN32 | *O. aries* |  | China |
| AY829389 | HY001 | *O. aries* | Hu | China |
| KF677094 | IVE_3 | *O. aries* | Ivesi | Turkey |
| KX344345 | J25 | *O. aries* |  | China |
| KX344366 | J47 | *O. aries* |  | China |
| HM236178 | kk12 | *O. aries* | Karakas | Turkey |
| DQ852283 | kk13 | *O. aries* | Karakas | Turkey |
| KF677028 | KRY_8 | *O. aries* | Karayaka | Turkey |
| JQ804989 | LH | *O. aries* |  | China? |
| HM236179 | mk4 | *O. aries* | Morkaraman | Turkey |
| KF677259 | MRK_43 | *O. aries* | Morkaraman | Turkey |
| KF677261 | MRK_46 | *O. aries* | Morkaraman | Turkey |
| KF677241 | MRK_9 | *O. aries* | Morkaraman | Turkey |
| KF677191 | NOR_8 | *O. aries* | Norduz | Turkey |
| KT148968 | OSC | *O. aries* | Oula sheep | China |
| AY829423 | PU_XW003 | *O. aries* | Small Tailed Han | China |
| AY829427 | PU_XW023 | *O. aries* | Small Tailed Han | China |
| KF677174 | SAK_17 | *O. aries* | Sakiz | Turkey |
| JN574156 | TKKRY06 | *O. aries* | Karayaka | Turkey |
| JN574163 | TKMOR10 | *O. aries* | Morkaraman | Turkey |
| HM236180 | mk3 | *O. aries* | Morkaraman | Turkey |
| HM236181 | mk9 | *O. aries* | Morkaraman | Turkey |
| DQ852280 | AW24 | *O. aries* | Awassi | Israel |
| HM236182 | AW25 | *O. aries* | Awassi | Israel |
| HM236183 | tj6 | *O. aries* | Tuj | Turkey |
| KF938360 | OG | *O. orientalis* | Asian mouflon | Kazakhstan |
| KF677291 | OGA_28 | *O. orientalis anatolica* | Asian mouflon | Turkey |
| KF677292 | OGA_29 | *O. orientalis anatolica* | Asian mouflon | Turkey |
| KF677293 | OGA_30 | *O. orientalis anatolica* | Asian mouflon | Turkey |
| KF677264 | OGA_1 | *O. orientalis anatolica* | Asian mouflon | Turkey |
| KF677265 | OGA_2 | *O. orientalis anatolica* | Asian mouflon | Turkey |
| KF677288 | OGA_25 | *O. orientalis anatolica* | Asian mouflon | Turkey |
| MG489885 | OAM_26 | *O. orientalis musimon* | European mouflon | Sardinia (Italy) |
| KR011772 | OAM_3a | *O. orientalis musimon* | European mouflon | Sardinia (Italy) |
| KR011774 | OAM_4 | *O. orientalis musimon* | European mouflon | Sardinia (Italy) |
| KR011775 | OAM_5 | *O. orientalis musimon* | European mouflon | Sardinia (Italy) |
| KR011776 | OAM_6 | *O. orientalis musimon* | European mouflon | Sardinia (Italy) |
| HM236184 | OAM_h1 | *O. orientalis musimon* | European mouflon | Germany |
| HM236185 | OAM_h2 | *O. orientalis musimon* | European mouflon | Germany |
| KF312238 | OGO | *O. orientalis ophion* | Asian mouflon | Cyprus |
| KR011780 | OGO_10 | *O. orientalis ophion* | Asian mouflon | Cyprus |
| KR011779 | OGO_9 | *O. orientalis ophion* | Asian mouflon | Cyprus |
| HM236186 | OV_h75 | *O. vignei* | Urial | Kazakhstan |
