# Supplementary material for: Identification of an ancestral haplotype in the mitochondrial phylogeny of the ovine haplogroup B
Source: PeerJ. 2019 Oct 22;7:e7895. doi: 10.7717/peerj.7895 (PMC6814065; doi:10.7717/peerj.7895)
Supplement: Table S2 [file peerj-07-7895-s002.docx]

| ACCESSION NUMBER | CODE | SPECIES | BREED | ORIGIN |
| --- | --- | --- | --- | --- |
| KY662385 | ASI | *O. aries* | Afshari | Iran |
| JX235859 | AWP2667 | *O. aries* | Awassi | Pakistan |
| HM236174 | cl122 | *O. aries* | Merino | Australia |
| KP228734 | GB68 | *O. aries* | Gangba | China |
| KU681221 | SFK_19 | *O. aries* | Suffolk | China |
| HE577849 | AIS | *O. aries* | Assaf | Israel |
| KF938340 | ANCR | *O. aries* | Andi | North Caucasian, Russia |
| KF938354 | FFL | *O. aries* | Finnsheep | Finland |
| KP228738 | GB71 | *O. aries* | Gangba | China |
| DQ097410 | HEM06_B | *O. aries* | Hemsin | Turkey |
| KF938328 | JZSC | *O. aries* | Jingzhong | Jinzhong, Shanxi, China |
| KF938353 | KGFL | *O. aries* | Kainuu grey | Finland |
| HM236176 | kk1 | *O. aries* | Karakas | Turkey |
| HM236177 | kk2 | *O. aries* | Karakas | Turkey |
| KF938358 | KLAR | *O. aries* | Kulunda | Altai, Russia |
| KF938351 | KNCR | *O. aries* | Karachai | North Caucasian, Russia |
| KU569707 | KPT | *O. aries* | Kail | China |
| KF938348 | KSEM | *O. aries* | Karakul | Southeast Europe, Moldova |
| KF938333 | KTCX | *O. aries* | Kazakh | Tekes County, Xingjiang, China |
| KF938346 | KVRR | *O. aries* | Kuibyshev | Volga Region, Russia |
| KU899145 | LLTC | *O. aries* | Lanzhou Large tailed | China |
| KF938357 | MCU | *O. aries* | Mountain carpathian | Ukraine |
| KF938344 | MSCA | *O. aries* | Mazekh | South-Caucasian, Azerbaijan |
| DQ320091 | OLL4 | *O. aries* | Finn Dorset | United Kingdom: Scotland |
| KF938352 | OVRR | *O. aries* | Oparin | Volga Region, Russia |
| KU899142 | PDC | *O. aries* | Poll Dorset | China |
| KF938347 | PKS | *O. aries* | Pramenka | Southeast Europe, Serbia |
| KP229032 | QH19 | *O. aries* | Qinghai Oula | China |
| KU899146 | SFC | *O. aries* | Suffolk | China |
| DQ903225 | SFK25 | *O. aries* | Skudde | China |
| KU899149 | TBC | *O. aries* | Tibetan | China |
| KF938343 | TSCR | *O. aries* | Tushin | South-Caucasian, Russia |
| KF938341 | ULUR | *O. aries* | Udmurtian local | Udmurtia, Russia |
| KF938350 | WPL | *O. aries* | Wrzosowka | Poland |
| DQ097423 | AKA06_C | *O. aries* | Akkaraman | Turkey |
| KU681187 | GT13 | *O. aries* | Tibetan | China |
| HM236178 | kk12 | *O. aries* | Karakas | Turkey |
| HM236179 | mk4 | *O. aries* | Morkaraman | Turkey |
| KT148968 | OSC | *O. aries* | Oula sheep | China |
| HM236180 | mk3 | *O. aries* | Morkaraman | Turkey |
| HM236181 | mk9 | *O. aries* | Morkaraman | Turkey |
| HM236182 | AW25 | *O. aries* | Awassi | Israel |
| MG407521 | Eis22 | *O. aries* |  | Egypt |
| DQ097430 | KAR15_C* | *O. aries* | Karayaka | Turkey |
| HM236183 | tj6 | *O. aries* | Tuj | Turkey |
| KF938360 | OG | *O. orientalis* | Asian mouflon | Kazakhstan |
| FJ936207 | OOKo1 | *O. orientalis* | Asian mouflon | Iran |
| EU365973 | OoaTk1 | *O. orientalis anatolica* | Asian mouflon | Turkey |
| EU365974 | OoaTk3 | *O. orientalis anatolica* | Asian mouflon | Turkey |
| FJ936185 | OOA3 | *O. orientalis anatolica* | Asian mouflon | Turkey |
| EU365986 | OoaTk2 | *O. orientalis anatolica* | Asian mouflon | Turkey |
| EU365987 | OoaTk4 | *O. orientalis anatolica* | Asian mouflon | Turkey |
| FJ936198 | OOG32 | *O. orientalis gmelini* | Asian mouflon | Turkey |
| FJ936190 | OOG2 | *O. orientalis gmelini* | Asian mouflon | Iran |
| FJ936202 | OOG8 | *O. orientalis gmelini* | Asian mouflon | Iran |
| FJ936186 | OOG11 | *O. orientalis gmelini* | Asian mouflon | Iran |
| FJ936189 | OOG15 | *O. orientalis gmelini* | Asian mouflon | Iran |
| FJ936203 | OOG9 | *O. orientalis gmelini* | Asian mouflon | Iran |
| EU366040 | OogAr1 | *O. orientalis gmelini* | Asian mouflon | Armenia |
| EU365979 | OogSn1 | *O. orientalis gmelini* | Asian mouflon | Iran |
| EU366009 | OogKh2 | *O. orientalis gmelini* | Asian mouflon | Iran |
| FJ936200 | OOG4 | *O. orientalis gmelini* | Asian mouflon | Iran |
| FJ936201 | OOG5 | *O. orientalis gmelini* | Asian mouflon | Iran |
| EU366016 | OgiAz1 | *O. orientalis isphahanica* | Asian mouflon | Iran |
| FJ936204 | OOI3 | *O. orientalis isphahanica* | Asian mouflon | Iran |
| EU365976 | OoiAz2 | *O. orientalis isphahanica* | Asian mouflon | Iran |
| FJ936209 | OOL1 | *O. orientalis laristanica* | Asian mouflon | Iran |
| FJ936210 | OOL2 | *O. orientalis laristanica* | Asian mouflon | Iran |
| EU365978 | OolBa1 | *O. orientalis laristanica* | Asian mouflon | Iran |
| MG489885 | OAM_26 | *O. orientalis musimon* | European mouflon | Sardinia, Italy |
| HM236184 | OAM_h1 | *O. orientalis musimon* | European mouflon | Germany |
| HM236185 | OAM_h2 | *O. orientalis musimon* | European mouflon | Germany |
| EU365977 | OomFr1 | *O. orientalis musimon* | European mouflon | France |
| EU365990 | OomFr2 | *O. orientalis musimon* | European mouflon | France |
| FR873149 | CYP_1665 | *O. orientalis ophion* | Asian mouflon | Cyprus |
| KF312238 | OGO | *O. orientalis ophion* | Asian mouflon | Cyprus |
| FJ936222 | OxV12 | *O. orientalis x vignei* | Hybrid mouflon/urial | Iran |
| FJ936224 | OxV14 | *O. orientalis x vignei* | Hybrid mouflon/urial | Iran |
| FJ936225 | OxV24 | *O. orientalis x vignei* | Hybrid mouflon/urial | Iran |
| FJ936227 | OxV32 | *O. orientalis x vignei* | Hybrid mouflon/urial | Iran |
| FJ936223 | OxV13 | *O. orientalis x vignei* | Hybrid mouflon/urial | Iran |
| FJ936226 | OxV25 | *O. orientalis x vignei* | Hybrid mouflon/urial | Iran |
| EU366045 | OvbPk1 | *O. vignei blanfordi* | Urial | Pakistan |
| HM236186 | OV_h75 | *O. vignei* | Urial | Kazakhstan |
| FJ936215 | OVA4 | *O. vignei arkal* | Urial | Kazakhstan |
| EU366042 | OvbTj1 | *O. vignei bochariensis* | Urial | Tajikistan |
| EU366043 | OvcPk1 | *O. vignei cycloceros* | Urial | Pakistan |
| EU366047 | OvpPk1 | *O. vignei punjabensis* | Urial | Pakistan |
| EU366049 | OvvPk1 | *O. vignei vignei* | Urial | Pakistan |
| EU366057 | OaUz1 | *O. ammon sevetzovi* | Argali | Uzbekistan |

.
