# Supplementary material for: Identification of an ancestral haplotype in the mitochondrial phylogeny of the ovine haplogroup B
Source: PeerJ. 2019 Oct 22;7:e7895. doi: 10.7717/peerj.7895 (PMC6814065; doi:10.7717/peerj.7895)
Supplement: Table S3 [file peerj-07-7895-s003.docx]

| SCIENTIFIC NAME | COMMON NAME | HPG | CODE | ORIGIN | GB # |
| --- | --- | --- | --- | --- | --- |
| *Addax nasomaculatus* | White screw horn antelope | - | - | - | NC_020674 |
| *Bos taurus* | Cattle | - | - | - | NC_006853 |
| *Boselaphus tragocamelus* | Nilgai antelope | - | - | - | NC_020614 |
| *Bubalus bubalis* | Water buffalo | - | - | - | AY488491 |
| *Connochaetes taurinus* | Blue wildebeest | - | - | - | NC_020699 |
| *Hippotragus niger* | Sable antelope | - | - | - | NC_020713 |
| *Kobus ellipsiprymnus* | Waterbuck | - | - | - | NC_020715 |
| *Kobus leche* | Red lechwe | - | - | - | NC_018603 |
| *Moschus moschiferus* | Siberian musk deer | - | - | - | JN632662 |
| *Oreamnos americanus* | Rocky Mountain goat | - | - | - | FJ207535 |
| *Pantholops hodgsoni* | Tibetan antelope | - | - | - | DQ191826 |
| *Redunca arundinum* | Southern reedbuck | - | - | - | NC_020794 |
| *Ovis ammon* | Argali | - | - | Kazakhstan | HM236188 |
| *Ovis aries* | Domestic sheep | A | HPG-A1 | Australia | HM236174 |
|  |  | A | HPG-A2 | Australia | HM236175 |
|  |  | B | HPG-B1 | Turkey | HM236176 |
|  |  | B | HPG-B2 | Turkey | HM236177 |
|  |  | C | HPG-C1 | Turkey | HM236178 |
|  |  | C | HPG-C2 | Turkey | HM236179 |
|  |  | D | HPG-D1 | Turkey | HM236180 |
|  |  | D | HPG-D2 | Turkey | HM236181 |
|  |  | E | HPG-E1 | Israel | HM236182 |
|  |  | E | HPG-E2 | Turkey | HM236183 |
| *Ovis orientalis musimon* | mainland Europe mouflon | B | Eur_M | Germany | HM236184 |
| *Ovis orientalis musimon* | Sardinian mouflon | B | Sar_M | Italy (Sardinia) | MG489885† |
| *Ovis canadensis* | Bighorn | - | - | Canada | JN181255 |
| *Ovis orientalis* | Asian mouflon | B | As_M | Kazakhstan | KF938360 |
| *Ovis orientalis ophion* | Cyprus mouflon | - | Cyp_M | Cyprus | KF312238 |
| *Ovis vignei* | Urial | - | - | Kazakhstan | HM236189 |

† Sequences obtained in the present study.
