# Supplementary figures and images for: Identification of an ancestral haplotype in the mitochondrial phylogeny of the ovine haplogroup B
Source: PeerJ. 2019 Oct 22;7:e7895. doi: 10.7717/peerj.7895 (PMC6814065; doi:10.7717/peerj.7895)

1

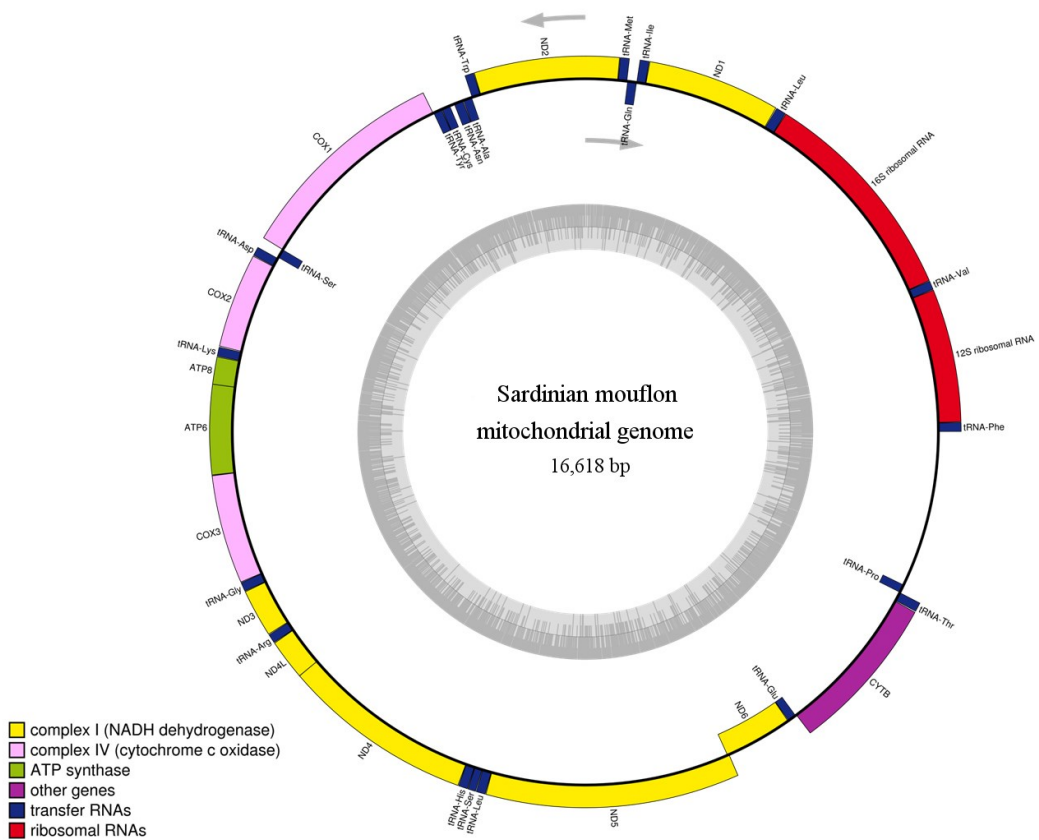

2

3

4

Supplement: Figure S1 — Arrows indicate the reading frame orientation of each strand. Different colours indicate functional gene categories as indicated in the legend. The inner circle is a graph depicting GC content across the genome (dark gray bars = percentage GC). [file peerj-07-7895-s004.pdf]

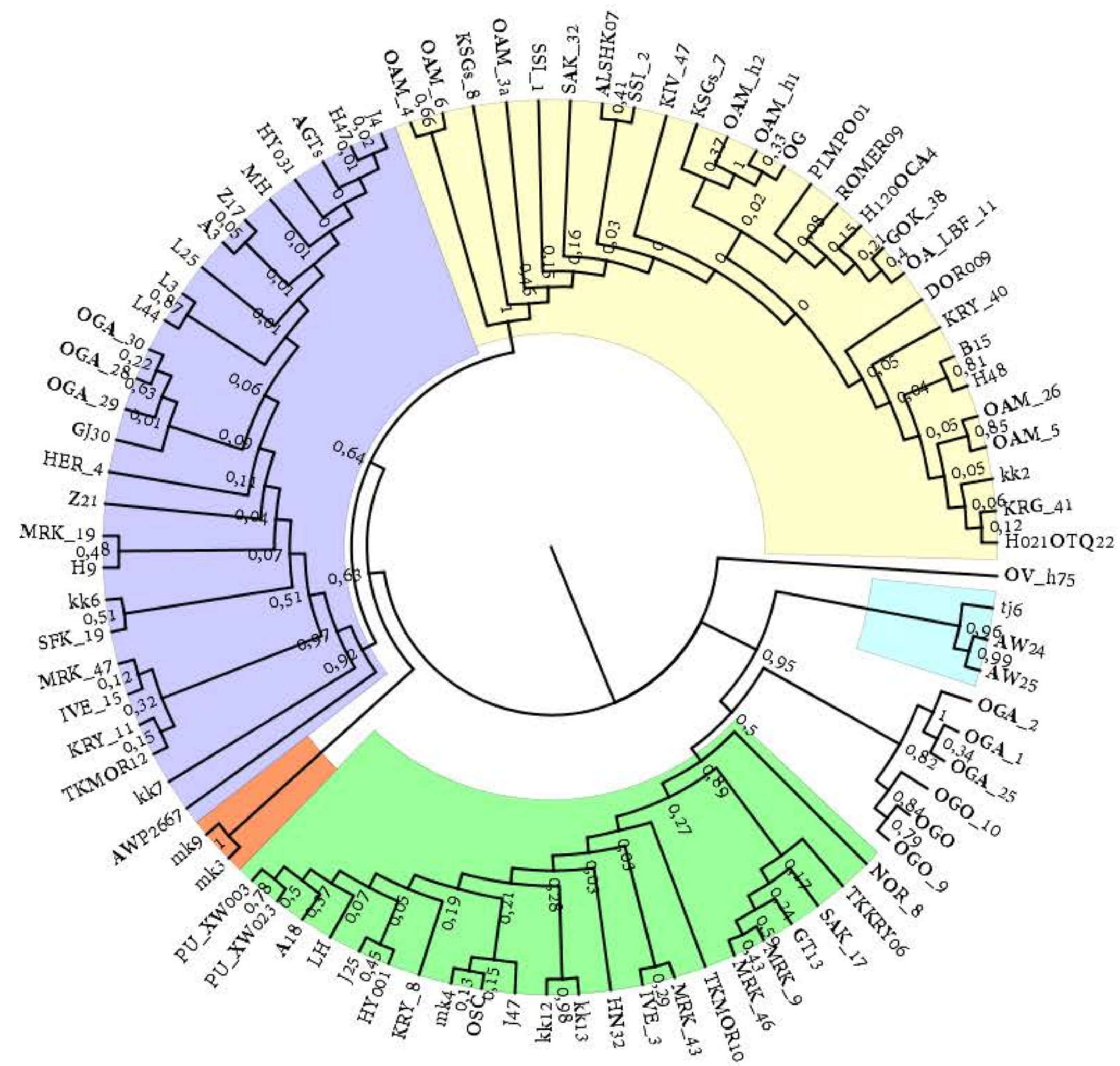

Supplement: Figure S2 [file peerj-07-7895-s005.pdf]

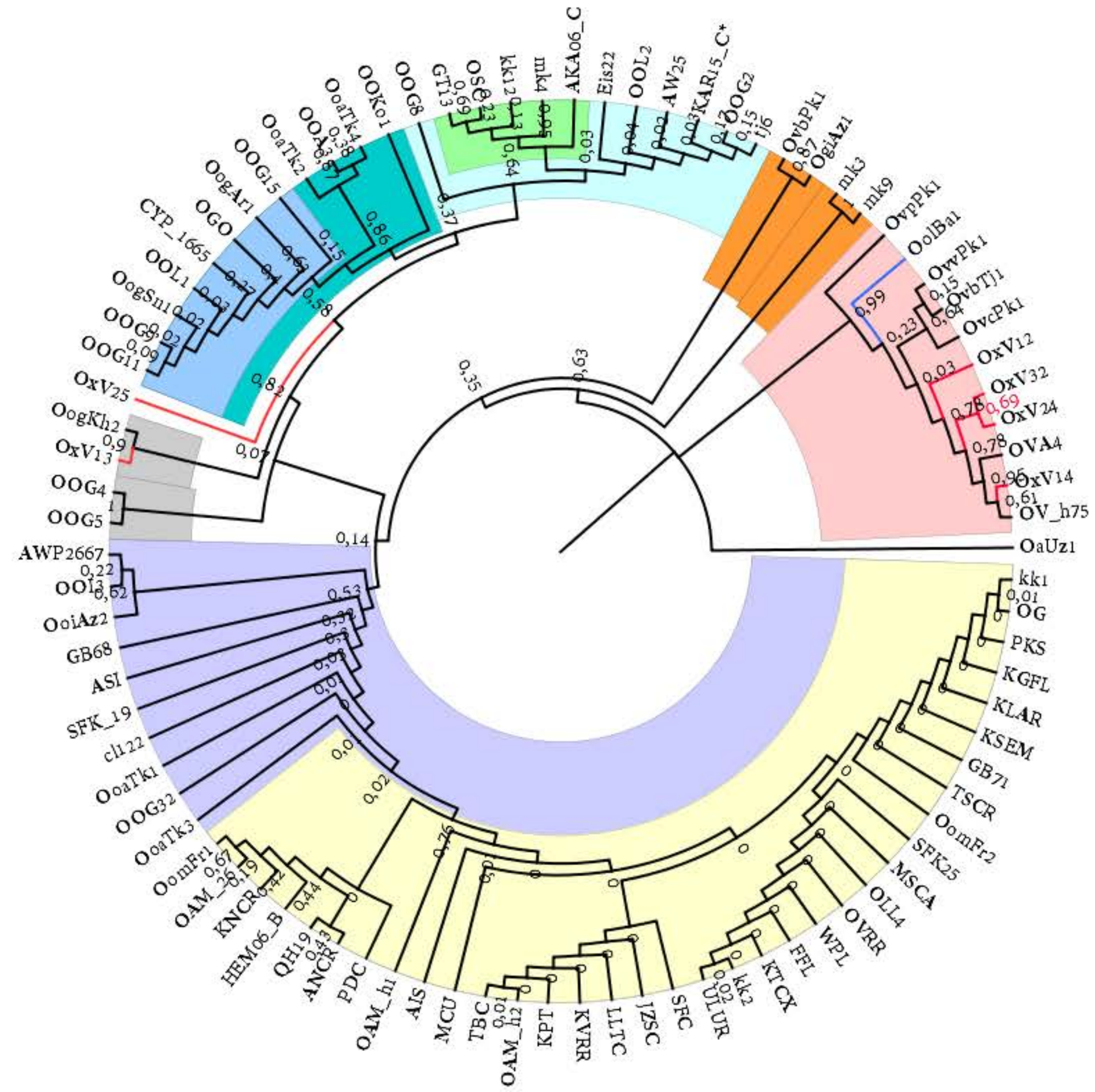

Supplement: Figure S3 [file peerj-07-7895-s006.pdf]

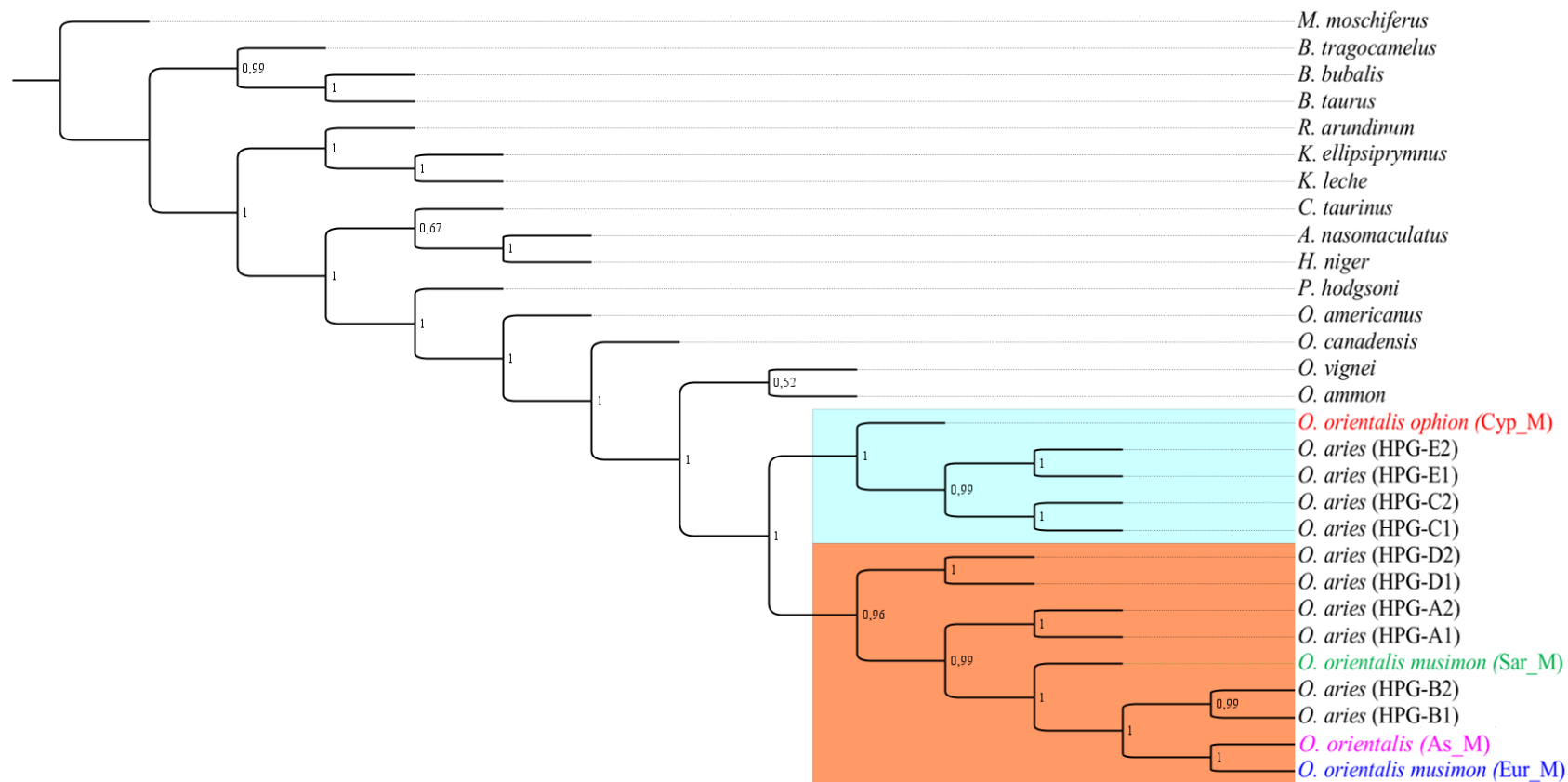

Supplement: Figure S4 — All nodes representing the domestic sheep and mouflon species radiation are supported with bootstrap values ≥ 0.96. The tree was rooted using Moschus moschiferus mtDNA genome [file peerj-07-7895-s007.pdf]
